# Preoptic activation induces a torpor-like hypothermic and hypometabolic state that is cerebroprotective

**DOI:** 10.1101/2025.10.24.684192

**Published:** 2025-10-25

**Authors:** Aizad Kamal, Juan Liu, Ernesto R Gonzales, Khairunisa Mohamad Ibrahim, Fan Zhang, Hannah E Skelton, Javier Kelly Cuenca, Carla Yuede, Gary J Patti, Leah P Shriver, Jin-Moo Lee, Aaron J Norris, Eric C Landsness

**Affiliations:** 1 Washington University School of Medicine, Department of Neurology; St. Louis, USA.; 2 Washington University School of Medicine, Department of Anesthesiology; St. Louis, USA.; 3 Washington University School of Medicine, Department of Psychiatry; St. Louis, USA.; 4 Washington University School of Medicine, Center for Biostatistics and Data Science; St. Louis, USA.; 5 Washington University School of Medicine, Department of Radiology; St. Louis, USA.; 6 Washington University School of Medicine, Mallinckrodt Institute of Radiology, Biophotonics Research Center; St. Louis, USA; 7 Washington University, Department of Chemistry; St. Louis, USA.; 8 Washington University, Center for Mass Spectrometry and Metabolic Tracing; St. Louis, USA.; 9 Washington University, Division of Nutritional Science and Obesity Medicine; St. Louis, USA.; 10 Washington University, Department of Biomedical Engineering; St. Louis, USA.; 11 Washington University, Department of Genetics and Medicine; St. Louis, USA.

## Abstract

Therapeutic hypothermia for stroke has been limited by shivering, increased metabolic demand, and poor patient tolerance. Engaging endogenous thermoregulatory circuits to lower body temperature may overcome these limitations and modulate metabolism, offering an integrated approach to cerebroprotection. Here, we show that chemogenetic activation of neurons in the preoptic area (POA) elicits a torpor-like state in mice, characterized by sustained hypothermia and hypometabolism. In an animal stroke model, this endogenous hypothermic state significantly reduced infarct volume and improved motor outcomes compared to controls, whereas maintaining normothermia attenuated these protective effects. To explore metabolic mechanisms contributing to this state, we performed untargeted metabolomic profiling 30 minutes after POA activation and identified coordinated shifts in nucleotide, phospholipid, and sphingolipid pathways. These rapid, temperature-dependent changes indicate a metabolically reprogrammed state that may enhance neuronal resilience during ischemic stress. Together, our findings suggest that POA-driven hypothermia confers cerebroprotection through specific metabolic adaptations with translational potential.

Therapeutic hypothermia has been shown to improve neurological outcomes and survival in patients with cardiac arrest^1,2^ and in neonates with hypoxic-ischemic encephalopathy^3^. Despite strong evidence from rodent pre-clinical models of stroke,^4–7^ clinical trials using therapeutic hypothermia after ischemic stroke have not demonstrated beneficial effects^8–11^. This lack of efficacy may be partially due to patient intolerance of cooling, which often necessitates sedation, as well as the significant metabolic and physiological stress associated with endogenous homeostatic responses, including shivering and counter-regulatory heat production^12^. In endotherms, including mice and humans, these responses involve considerable energy expenditure to oppose reductions in body temperature^12,13^, potentially masking or attenuating beneficial metabolic effects of hypothermia. Targeting specific neuronal populations that regulate thermal homeostasis could overcome these barriers by reducing unwanted physiological stress responses. Moreover, understanding the metabolic adaptations driven by neural circuit-mediated hypothermia may uncover novel and impactful mechanisms with relevance for cerebroprotection.

Within the preoptic area, specific neuronal populations respond to changes in environmental temperature or food deprivation by modulating body temperature and global metabolism^14–17^. Recent studies have further identified a subpopulation of POA neurons expressing the kappa opioid receptor (KOR) that plays a critical role in controlling these thermoregulatory and metabolic processes. Activation of these KOR-expressing neurons (POA^KOR+^) induces a profound hypothermic and hypometabolic state, highlighting a potential endogenous mechanism for temperature and metabolic modulation^18,19^. Importantly, KOR-expressing neurons are also present in the human POA, suggesting that analogous pathways may be evolutionarily conserved^20,21^. By selectively activating these endogenous circuits, it may be possible to achieve a controlled, torpor-like reduction in body temperature and modulation of metabolism, potentially circumventing the physiological stress response typically associated with traditional hypothermia techniques.

In this study, we sought to determine whether activating endogenous thermoregulatory circuits in the POA can induce a torpor-like hypothermic state that confers cerebroprotection in the setting of acute ischemia. We further aimed to explore whether this protection may depend on hypothermia itself or might also involve temperature-independent metabolic effects of neural activation. To explore these questions, we combined chemogenetic manipulation of POA neurons with a model of transient cerebral ischemia and performed comprehensive untargeted metabolomic profiling to examine metabolic adaptations associated with this protective state. Our findings suggest that endogenous POA activation engages hypothermia-associated metabolic reprogramming and supports brain resilience, offering a potentially translatable, circuit-driven strategy that extends beyond conventional cooling approaches.

## RESULTS

### Activation of POA^KOR+^ neurons induces long-lasting hypothermia, reduces stroke size, and improves behavioral outcomes in a temperature-dependent manner

**Figure 1:**
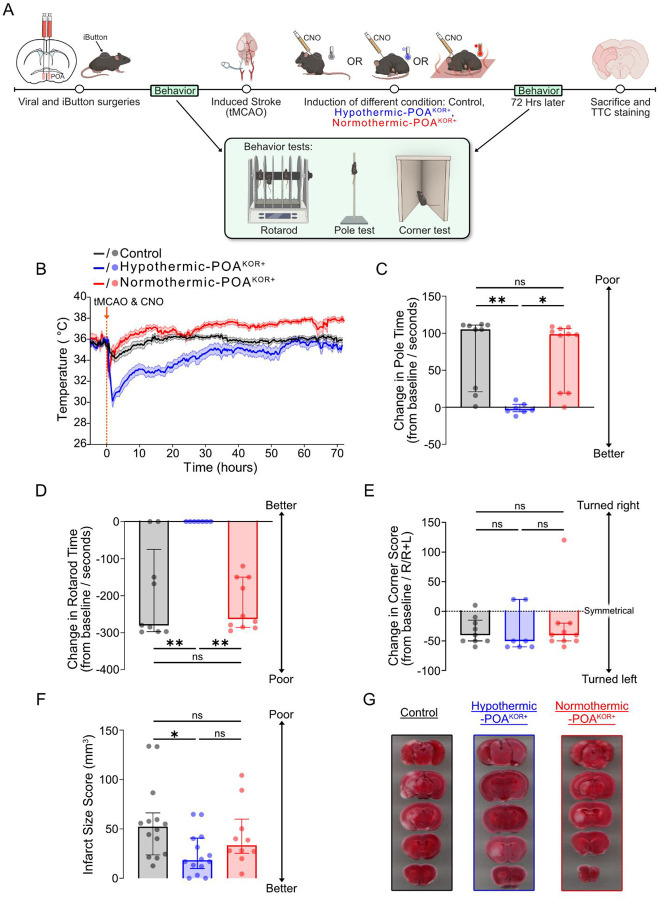
Activation of POA^KOR+^ neurons induces a hypothermic response for 24 hours and is associated with improved outcomes following stroke. (A) Experimental timeline: KOR-Cre mice underwent viral injection followed by iButton temperature logging implantation, baseline behavioral testing, and a 30-minute transient middle cerebral artery occlusion (tMCAO), with CNO (clozapine-N-oxide) injection to induce a hypothermic and hypometabolic state: control (N = 17), hypothermic- POA^KOR+^ (N = 16), or normothermic - POA^KOR+^ (N = 10). After 72 hours, animals were re-assessed through behavioral testing and histology. (B) Core body temperature curves for the control, hypothermic-POA^KOR+^,and normothermic-POA^KOR+^ groups over 72 hours following stroke. (C) Change in pole time, measured as the difference between baseline and 72 hours post-stroke for all groups. *P < 0.05 and **P < 0.01 by post-hoc Dunn’s tests. (D) Change in rotorod time, quantified as the difference between baseline and 72 hours post-stroke, comparing all groups. **P < 0.01 by post-hoc Dunn’s tests. (E) Corner test asymmetry, calculated as the number of right turns expressed as a percentage of the total number of turns (right + left), for all groups. (F) Quantification of infarct size using direct measurement, comparing the hypothermic- POA^KOR+^, control groups and normothermic-POA^KOR+^ groups. *P < 0.05 by Tukey’s multiple comparison test. (G) Representative TTC (2,3,5-triphenyltetrazolium chloride) stained coronal sections from 3 individual mice, showing unstained infarct regions. Note: behavioral analyses (C–E) were performed on subsets of animals: Hypothermic-POA^KOR+^ (N = 7), Normothermic- POA^KOR+^ (N = 10), and Control (N = 9).

Given the limited success of external cooling in clinical stroke trials^8,9^, we sought to determine whether activation of endogenous neural circuits in the POA might induce a torpor-like hypothermic state that may confer cerebroprotection. We hypothesized that such activation could protect the brain through sustained hypothermia and associated metabolic changes. To test this hypothesis, we used chemogenetic activation of POA^KOR+^ neurons and compared cerebroprotection after ischemic stroke under conditions that either allowed hypothermia or maintained normothermia. To explore whether protection may depend on hypothermia itself or involve temperature-independent effects of POA^KOR+^ activation, we designed three experimental groups following ischemic stroke: (1) control mice injected with a control virus, housed at a thermoneutral temperature (30°C), (2) POA^KOR+^-activated mice housed at 30°C, which reliably entered a torpor-like hypothermic state, termed “hypothermic-POA^KOR+^ ” (Supplementary Fig. S1); and (3) POA^KOR+^ activated mice housed at an at an elevated ambient temperature (36°C), where hypothermia was minimized, termed “normothermic-POA^KOR+^ ”. All mice underwent 30-minute transient middle cerebral artery occlusion (tMCAO), followed by behavioral testing and infarct quantification at 72 hours. Core body temperature was monitored continuously throughout the experiment, enabling us to assess the role of temperature in mediating the effects of POA^KOR+^ activation on stroke outcomes (Fig. 1A).

Core body temperature trajectories revealed a sustained and selective hypothermic response in the hypothermic-POA^KOR+^ group housed at 30°C. Within the first 12 hours post-activation, this group exhibited a mean temperature reduction of 3.35°C (95% CI: 2.50–4.19°C), compared to 0.55° C (95% CI: 0.05–1.06°C) in the control group and 1.73°C (95% CI: 0.53–2.92°C) in the normothermic-POA^KOR+^-activated group. One-way ANOVA revealed a significant difference in temperature reduction across groups (F(2,40) = 16.54, *P* < 0.0001, R^2^ = 0.4526), with Tukey’s multiple comparisons test showing a significant difference between hypothermic-POA^KOR+^ and control (*P* < 0.0001), and between hypothermic-POA^KOR+^ and normothermic-POA^KOR+^(*P* = 0.0173), but not between control and normothermic-POA^KOR+^(*P* = 0.1009). (Fig. 1B). Temperatures in the hypothermic-POA^KOR+^ group gradually returned toward baseline after 24 hours, while the other two groups maintained relatively stable temperatures throughout the monitoring period. These findings show that only POA^KOR+^ activation under permissive thermal conditions produces a sustained hypothermic state, allowing us to assess whether the cerebroprotective effects of POA^KOR+^ activation may depend on hypothermia.

At 72 hours post-stroke, POA^KOR+^ activation under hypothermic conditions was associated with preserved motor function, whereas normothermic-POA^KOR+^ mice showed motor impairment comparable to control. In the pole test, hypothermic-POA^KOR+^ mice showed minimal change from baseline (median change = −4.0 sec, 95% CI [−12, 10]), in contrast to control (median change = 105.0 sec, 95% CI [16, 111] and normothermic-POA^KOR+^ groups (median change = 98.5 sec, 95% CI [19, 108]; Kruskal–Wallis test; *P* = 0.0010 Fig. 1C). A similar pattern emerged in the rotarod test, hypothermic-POA^KOR+^ mice maintained performance close to baseline (median change = 0.0 sec, 95% CI [0, 0]), while control mice declined significantly (median change = −280.0 sec, 95% CI [0, −297]) and normothermic-POA^KOR+^ mice also performed poorly (median change = −262.5 sec, 95% CI [–150, −287]; Kruskal–Wallis test; *P* = 0.0021; Fig. 1D). All three groups demonstrated persistent post-stroke asymmetry on the corner test, with no significant differences among groups (hypothermic-POA^KOR+^ median = −50.0%, 95% CI [–60, 20]; control median = −40.0%, 95% CI [−50, −10]; normothermic-POA^KOR+^ median = −40.0%, 95% CI [–50, −20]; Kruskal–Wallis test; *P* = 0.5872; Fig. 1E).

Infarct size was markedly reduced in the hypothermic-POA^KOR+^ group (mean = 24.82 mm^3^, 95% CI [12.52, 37.13]) compared to controls (mean = 56.69 mm^3^, 95% CI [34.78, 78.61]), and trended smaller than the normothermic-POA^KOR+^ group (mean = 42.91 mm^3^, 95% CI [20.70, 65.13]; ANOVA test; *P* = 0.0334; Fig. 1F, G). Mortality rates at 72 hours were 17.65% in the control group (3/17), 18.75% in the hypothermic-POA^KOR+^ group (3/16), and 0% in the normothermic-POA^KOR+^ group (0/10); Fisher’s Exact test; *P* = 0.4702), suggesting that differences in infarct size and behavioral outcomes are not likely explained by survivorship bias. Body weight loss was comparable across groups: hypothermic-POA^KOR+^ mice lost a median of 15% (95% CI: −5.7% to −3.6%), control mice lost 13.55% (95% CI: −7.4% to −1.4%), and normothermic-POA^KOR+^ mice lost 13.75% (95% CI: −6.4% to −1.1%) (Kruskal–Wallis test; *P* = 0.7237).

**Figure 2:**
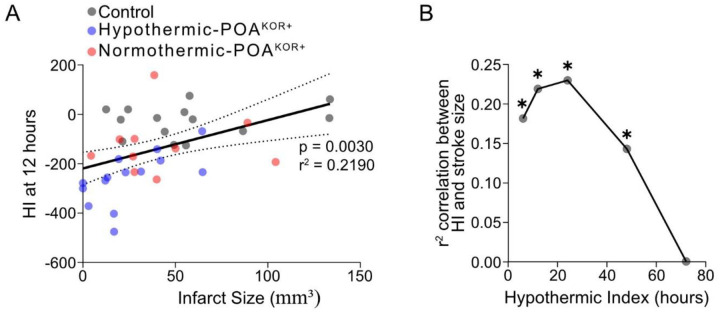
Degree of hypothermia correlates to direct stroke size. (A) The hypothermic index (HI) was calculated based on the amount of time and depth of hypothermia during the 12-hour period following stroke, and this was correlated with direct infarct size. (B) The correlation between HI calculated for 6, 12, 24, 48, and 72 hours and stroke size are shown, with r^2^ values representing the strength of the relationship. *P < 0.05

We next investigated whether the extent of hypothermia was associated with infarct size, using the hypothermic index, defined as the area under the temperature curve relative to baseline. A significant correlation was observed between the hypothermic index and stroke size at 6 hours (r^2^ = 0.18, *P* = 0.008), 12 hours (r^2^ = 0.22, *P* = 0.003; Fig 2A), 24 hours (r^2^ = 0.23, *P* = 0.002) and 48 hours (r^2^ = 0.143, *P* = 0.019) post-stroke, but not 72 hours (Fig 2B). Thus, greater depth and duration of hypothermia is associated with smaller infarct sizes, and the therapeutic efficacy diminishes beyond 48 hours.

### Activation of POA^KOR+^ neurons rapidly alters brain metabolism

**Figure 3:**
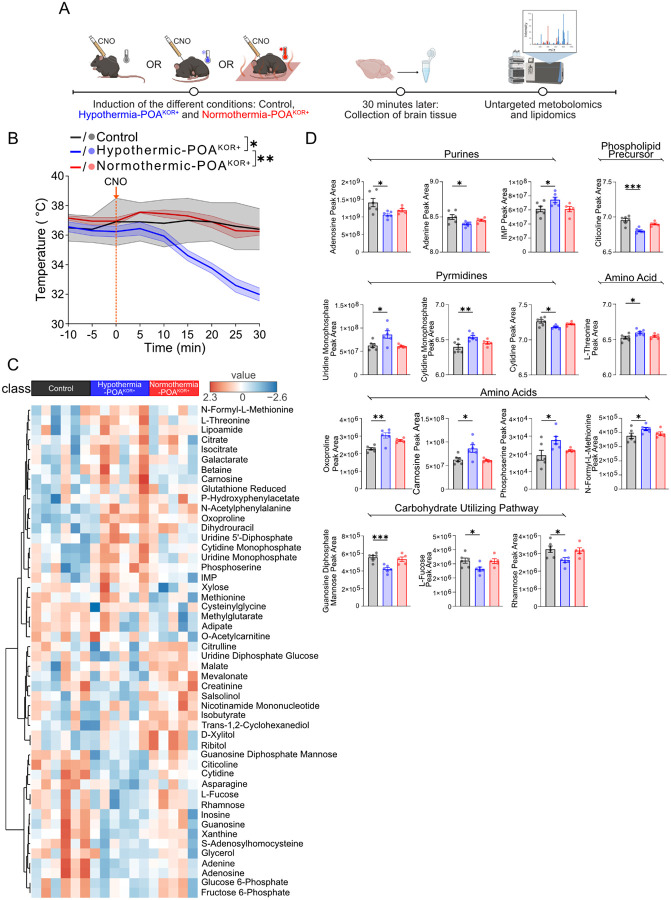
Early metabolomic changes following POA^KOR+^ activation under hypothermic and normothermic conditions. (A) Experimental design for untargeted metabolomics and lipidomics. Three groups were analyzed: hypothermic-POA^KOR+^ mice housed at room temperature (~22°C; N=6), normothermic-POA^KOR+^ mice housed at 36°C (N=5), and controls housed at room temperature (N=6). (B) Core body temperature traces in the 30 minutes following CNO injection for each experimental group. *P < 0.05, **P < 0.01 by Dunn’s post-hoc t-tests. (C) Heatmap of metabolite abundance across conditions for the top 50 polar metabolites showing the greatest separation between groups. Metabolite intensities were log10-transformed and Z-score normalized prior to visualization. Red indicates higher relative abundance and blue indicates lower relative abundance for each metabolite. (D) Fifteen metabolites with uncorrected *t*-tests *P < 0.1, **P < 0.05, ***P < 0.01 in the hypothermic- POA^KOR+^ group (relative to control and normothermic- POA^KOR+^ groups). Abbreviation: IMP (inosine monophosphate).

Given that POA^KOR+^ activation was cerebroprotective only under hypothermic conditions, we hypothesized that hypothermia induces specific metabolic adaptations may support this effect. To capture the earliest, circuit-driven metabolic changes before ischemic injury or inflammation could confound results, we collected whole-brain tissue 30 minutes after POA^KOR+^ activation. We compared three groups: (1) hypothermic-POA^KOR+^ mice, housed at room temperature (~22°C), that developed hypothermia following activation; (2) normothermic-POA^KOR+^ mice, housed at an elevated ambient temperature (36°C), in which hypothermia was prevented; and (3) control mice injected with control virus and housed at room temperature (Fig 3A). Core body temperature measurements confirmed that only the hypothermic-POA^KOR+^ group exhibited a significant drop in temperature post-activation(median temperature drop of −4.05°C; 95% CI: −6.300 – −2.700). By contrast, the normothermic-POA^KOR+^ (median temperature drop of 0.50°C; 95% CI: −1.000 – 3.900) and control groups (median temperature drop of −0.200°C; 95% CI: −0.600 – 0.400) maintained stable temperatures (Kruskal–Wallis test, H(2) = 10.70, *P* = 0.0005; Fig. 3B). This design provided a framework to distinguish temperature-dependent effects of POA activation from changes due to neural stimulation alone.

To explore how POA^KOR+^ activation alters brain metabolism under hypothermic versus normothermic conditions, we first examined global metabolic patterns using heatmaps of the top 50 polar metabolites that best distinguished the experimental groups (Fig. 3C). In control animals, metabolic profiles were relatively uniform, consistent with minimal metabolic activation in the absence of neural stimulation. In contrast, hypothermic-POA^KOR+^ mice exhibited a distinct and coordinated shift across multiple metabolite classes, suggesting a temperature-sensitive metabolic response to circuit activation. Interestingly, normothermic-POA^KOR+^ mice displayed profiles either resembling controls or shifting in the opposite direction from hypothermic-POA ^KOR+^ mice. This pattern suggests that preventing hypothermia may alter the typical metabolic program induced by POA^KOR+^ activation, underscoring the role of temperature in shaping brain metabolic state.

Following our heatmap analysis, we next performed statistical comparisons to identify specific polar metabolites selectively altered by circuit-driven hypothermia. We compared the hypothermic-POA^KOR+^ group to both normothermic-POA^KOR+^ and control groups, initially screening for metabolites with uncorrected *P*-values < 0.1 to avoid prematurely excluding potentially meaningful changes. This exploratory analysis identified 15 polar metabolites that were selectively altered in the hypothermic-POA^KOR+^ group, but not in normothermic or control animals. Pathway enrichment analysis revealed that these metabolites were predominantly involved in purine and pyrimidine metabolism, phospholipid biosynthesis, and carbohydrate-utilizing pathways (Fig. 3D). These pathways include processes such as nucleotide salvage, membrane phospholipid turnover, and biosynthetic adaptations to cellular stress, all of which have been associated with resilience under ischemic conditions^29–32^. To identify the most robust changes, we then applied false discovery rate (FDR) correction (threshold < 0.1) across all measured metabolites. Three metabolites: citicoline, guanosine diphosphate mannose, and oxoproline—remained significant after correction. While the remaining twelve metabolites did not meet FDR significance, they showed directional trends consistent with a coordinated response to hypothermia and are included in Fig. 3D for reference. Together, these data show that circuit-driven hypothermia is associated with coordinated changes, including pathways related to nucleotide salvage, membrane remodeling, and stress response.

**Figure 4:**
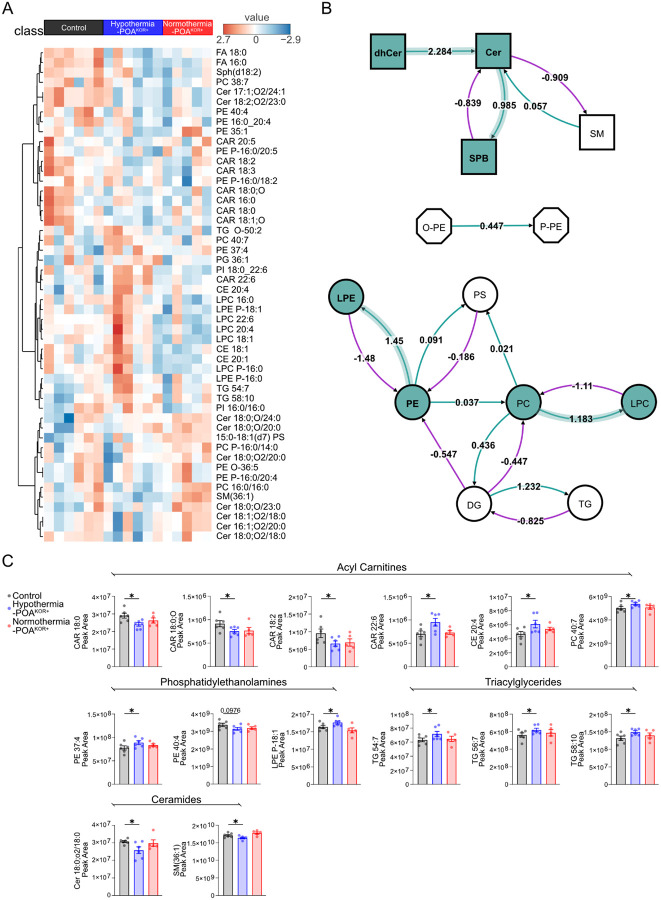
Temperature-dependent lipidomic changes following POA^KOR+^ activation. A) Heatmap of lipid abundance across conditions for the top 50 lipids showing the greatest separation between the hypothermic (N=6), normothermic (N=5) and control (N=6) conditions. (B) Pathway analysis for lipid metabolism. Nodes represent different lipid classes. Edges indicate transformations between lipid species, with numbers representing Z-score change in lipid conversion. Cyan nodes and arrows represent increased conversion in the hypothermic- POA^KOR+^ group compared to control, but not normothermic- POA^KOR+^ using a threshold of |Z-score| > 0.95. White nodes and unshaded arrows indicate lipids with no significant change in conversion. (C) Fourteen individual lipids that were significantly different (uncorrected *P < 0.1) in the hypothermic- POA^KOR+^ condition relative to control, but not the normothermic condition. Lipid class abbreviations: dhCer (Dihydroceramide), Cer (Ceramide), SBP (Sphingosine), SM (Sphingomyelin), PE (Phosphoethanolamine), PS (Phosphoserine), PC (Phosphocholine), DG (Diacylglycerol), TG (Triacylglycerol), FA (Fatty Acid), Sph (Sphinganine), CAR (Carnitine), PE P (Plasmalogen Phosphoethanolamine), PG (Phosphoglycerol), PI (Phosphatidylinositol), CE (Cholesteryl Ester), LPC (Lysophosphatidylcholine), LPE (Lysophosphatidylethanolamine), O-PE (Ether-linked Phosphoethanolamine), P-PE (Plasmenyl-Phosphoethanolamine).

Next, untargeted lipidomics was performed on whole-brain extracts from the same experimental groups. We first generated a heatmap of the top 50 lipids showing the greatest variation across conditions (Fig. 4A). As observed with polar metabolites, hypothermic-POA^KOR+^ mice exhibited widespread lipid changes, while normothermic-POA^KOR+^ and control samples showed more stable profiles. We then performed pathway-level analysis to identify lipid pathways selectively altered in the hypothermic-POA^KOR+^ group compared to controls but unchanged in normothermic conditions (Fig. 4B). This analysis highlighted lipid pathways selectively altered in the hypothermic- POA^KOR+^ group, including sphingolipid processing (e.g., conversion of dihydroceramides to ceramides, increased sphingoid base production), phosphatidylcholine breakdown, and lysophospholipid remodeling. These pathways are broadly associated with membrane remodeling, stress response, and cellular energetics, and have been previously linked to resilience mechanisms during ischemic stress^33–41^. Following pathway analysis, we screened individual lipid species for differences between groups using an initial uncorrected *P*-value threshold of < 0.1. Although no individual lipids met significance after FDR correction, several lipid classes, including acylcarnitines, cholesteryl esters, ceramides, sphingomyelins, phosphatidylethanolamines, and triglycerides, exhibited directional changes that were selectively enriched in the hypothermic condition (Fig. 4C). Together, these findings indicate that circuit-driven hypothermia is associated with temperature-dependent lipid remodeling, which have also been implicated as cerebroprotective under conditions of ischemic stress.

## DISCUSSION

Our study shows that activating POA^KOR+^ neurons induces a torpor-like hypothermic, hypometabolic state that reduces infarct size and preserves motor function after ischemic stroke, primarily when hypothermia is allowed to develop. Under these conditions, we observed temperature-dependent changes in brain metabolism, characterized by shifts in nucleotide salvage, phospholipid remodeling, and sphingolipid pathways. These findings suggest that engaging endogenous thermoregulatory circuits may engage a metabolically coordinated state associated with protection, offering a mechanistically distinct strategy from conventional external cooling. These initial findings establish a foundation for future studies aimed at understanding the mechanisms of circuit-driven hypothermia and exploring its potential to improve cerebroprotection in clinical settings.

To investigate potential mechanisms by which circuit-driven hypothermia might confer cerebroprotection, we examined early brain metabolic responses following POA^KOR+^ activation. Under hypothermic conditions, our untargeted metabolomics analysis revealed coordinated shifts across several pathways, including nucleotide salvage, phospholipid remodeling, and sphingolipid metabolism, while these shifts were largely absent or directionally different in normothermic animals. These findings support the idea that hypothermia may engage specific biochemical processes rather than merely lowering core temperature.

These pathways have been previously associated with cellular resilience during ischemic stress. For example, enhanced purine and pyrimidine salvage could support energy conservation and macromolecular repair by maintaining ATP levels and nucleotide pools^42–45^. Phospholipid remodeling, evidenced by changes in citicoline (a molecule linked to cerebroprotection^46–50^) and lysophosphatidylcholine levels, may reflect adaptive changes in membrane structure and signaling, which are critical for neuronal survival and inflammation resolution^46,48–52^. Meanwhile, alterations in sphingolipid metabolism suggest a potential shift away from pro-apoptotic ceramides toward bioactive lipids with protective roles^53,54^, though definitive confirmation will require direct measurement of downstream mediators like sphingosine-1-phosphate. While these observations remain correlative, they outline a plausible biochemical framework through which circuit-driven hypothermia might reduce ischemic injury. Importantly, delineating these metabolite and pathway changes may reveal candidate targets for pharmacologic intervention, potentially enabling new strategies that mimic or augment the protective effects of circuit-driven hypothermia. This work sets the stage for future studies employing metabolic flux analysis to validate these pathways, quantify their activity, and test whether neural circuits actively coordinate protective metabolic states. Such insights will be essential for determining how circuit-driven hypothermia might be leveraged to improve outcomes in stroke and other neurological injuries.

Our metabolomic findings suggest that POA^KOR+^ activation elicits a hypothermic state that reflects more than a simple reduction in core temperature, instead engaging metabolic pathways with potential relevance for brain protection. These findings align with prior work showing that defined POA circuits can influence systemic metabolism, including glucose regulation and energy expenditure, through mechanisms not strictly dependent on thermoregulation^14,55^. At the same time, maintaining normothermia during POA-induced hypothermia found that the associated glucose hypometabolism was attenuated, indicating that reduced body temperature also contributes to the metabolic reprogramming observed after POA activation^56^. Taken together, these observations highlight that circuit-driven hypothermia coordinates both temperature-dependent and temperature-independent metabolic programs, a feature not replicated by traditional cooling approaches. This distinction is important because conventional cooling methods, despite strong preclinical promise, have shown limited clinical efficacy due to counter-regulatory responses such as shivering which can increase metabolic demand.^12,13^ By engaging endogenous thermoregulatory circuits, our approach offers a more integrated means of reducing both temperature and modulating metabolism, potentially bypassing the physiological barriers that undermine conventional hypothermia. These observations are consistent with recent work showing that modulation of central pathways, particularly within the POA, can be sufficient to achieve cerebroprotection^57^, highlighting the translational potential of circuit-driven thermoregulatory strategies^57,58^.

Given their central role in regulating both metabolism and temperature, endogenous thermoregulatory circuits represent a mechanistically grounded target for clinical translation. A longstanding challenge has been whether torpor-like states can be induced in non-hibernating species such as rats or humans. Recent studies using focused ultrasound have shown that such states can be evoked in rats, overcoming a long-standing species barrier and supporting potential clinical relevance.^59^ The presence of KOR-expressing neurons in the human POA indicates that these thermoregulatory circuits are conserved, supporting their potential as translational targets for inducing protective hypothermia^20,21^. Our findings extend this work by showing that POA^KOR+^-mediated hypothermia engages a coordinated physiological state accompanied by distinct shifts in metabolic pathways, including nucleotide, phospholipid, and sphingolipid metabolism. Together, targeting conserved thermoregulatory circuits in the POA, combined with metabolomic profiling may provide a blueprint for inducing, monitoring, and refining protective hypothermic states in translational contexts.

In our study, both the depth and duration of hypothermia during the first 48 hours were correlated with reduced infarct size, suggesting that in our model, the magnitude and persistence of cooling may contribute to cerebroprotection. Specifically, a higher cumulative hypothermic index over the first 6 to 48 hours (reflecting moderate hypothermia, 29–34°C) was associated with smaller infarcts, consistent with previous studies supporting the idea that sustained cooling may enhance cerebroprotection^7,60^. However, other investigations have reported protective effects even with milder or shorter-duration hypothermia, particularly when initiated soon after stroke onset^61,62^. Collectively, these varied findings highlight the need for future studies to define the optimal timing and depth of hypothermia for stroke treatment ideally in ways that maximize protection while maintaining physiological compatibility.

While our normothermia condition successfully prevented hypothermia and allowed us to test its necessity for POA^KOR+^-mediated cerebroprotection, it also introduced two variables: chemogenetic activation and elevated ambient temperature (36°C), each of which could independently influence stroke outcomes. This condition approximates the temperature profile of controls while maintaining neural circuit activation, offering a mechanistically informative contrast. Although infarct size was numerically lower in the normothermic- POA^KOR+^ group compared to controls, behavioral outcomes were unimproved, and metabolic reprogramming was modulated. These findings suggest that hypothermia contributes substantially to the protective phenotype, though temperature-independent metabolic mechanisms may also be involved. Indeed, evidence suggests that POA-induced alteration in metabolism is independent of its effects on body temperature^55,56^. Future studies could help clarify the contributions of ambient temperature alone by including additional controls, such as non-activated mice housed at 36°C. Moreover, time-course metabolomic analyses at 6, 12, 24 and 48 hours post-activation could determine whether POA^KOR+^ activation under normothermic conditions triggers a transient or divergent metabolic trajectory. Cell-type–specific profiling may also help determine whether distinct components of the neurovascular unit engage temperature-independent metabolic programs that are activated, but may not be sufficient for protection.^61,63,64^ These follow-up experiments, while beyond the scope of the current study, will be important for refining our understanding of how neural circuits coordinate temperature and metabolism, including potential temperature-independent mechanisms, to protect the brain.

Another important factor relates to the timing of our metabolomic profiling. Our metabolomic profiling was performed 30 minutes after POA^KOR+^ activation and in the absence of stroke or reperfusion injury, which precludes direct assessment of how these metabolic changes interact with ischemic tissue. However, this design also provides a key advantage: it captures the immediate biochemical effects of POA^KOR+^ activation before secondary processes such as inflammation, tissue injury, or catabolic stress can develop. By focusing on this early phase, our data offers a clearer view of temperature-dependent metabolic reprogramming driven by thermoregulatory activation and highlight candidate pathways that may contribute to cerebroprotection. Future studies incorporating post-stroke, time-course metabolomics will be important for determining the durability and functional significance of these early metabolic shifts, as well as their relevance for translational applications.

Taken together, our findings suggest that engaging endogenous thermoregulatory circuits to induce a torpor-like state offers a metabolically coordinated and mechanistically grounded approach to cerebroprotection. By identifying metabolic pathways altered during this state, including nucleotide salvage, phospholipid remodeling, and sphingolipid signaling, our study highlights potential targets that may complement or enhance traditional cooling strategies. This perspective is particularly relevant given the physiological and logistical challenges faced by clinical hypothermia trials. Future research will be needed to clarify how these metabolic shifts contribute to protection and to determine whether circuit-driven hypothermia, or targeted modulation of its downstream pathways, can inform the development of precise, metabolism-guided interventions for stroke and related neurological conditions.

## METHODS

### Experimental Design

#### Neural circuit activation to induce a hypothermic and hypometabolic state akin to torpor

A total of 43 mice were used in this experiment, with 20 males and 23 females. Mice received a stereotactic Designer Receptors Exclusively Activated by Designer Drugs (DREADDs) injection into the bilateral POA 4 weeks prior to stroke induction to allow for receptor expression. Three weeks later, an iButton (temperature monitoring sensor) was surgically implanted, followed by a one-week recovery period before tMCAO surgery. Baseline behavioral tests, including the rotarod, pole test, and corner test, were conducted on the same day before stroke induction to establish baseline performance. Stroke was induced via tMCAO for 30 minutes. Immediately following stroke induction, mice were administered an intraperitoneal (IP) injection of CNO, 0.5 mg/kg. There were three experimental groups: (1) control mice injected with a control virus, housed at a thermoneutral temperature (30°C), (2) POA^KOR+^-activated mice housed at 30°C, which reliably entered a torpor-like hypothermic state, termed “hypothermic-POA^KOR+^ ”; and (3) POA^KOR+^ activated mice housed at an at an elevated ambient temperature (36°C), in which hypothermia was actively suppressed, termed “normothermic-POA^KOR+^ ”. The control and hypothermic-POA^KOR+^ groups were then single-housed and placed in an incubator maintained at the thermoneutral temperature of 30°C for 72 hours. The normothermic- POA^KOR+^ group was placed in an incubator maintained at 36°C for 72 hours. The mice were monitored with ad-libitum access to food and water and daily weighing. If any mouse lost >20% of the body weight, they were euthanized per the Washington University Animal Studies Committee protocol. At 72 hours post-surgery, behavioral tests were repeated to evaluate recovery. Finally, cerebral blood flow was assessed, and the mice were euthanized for brain collection, TTC staining, and preservation in formalin solution for stroke size analysis.

### Metabolomics

A total of 17 mice consisting of 8 males and 9 females were used in this study. Mice were injected with the DREADDs virus, followed by the placement of a temperature programmable transponder after a recovery period of 4 weeks. Before the experiment, each mouse was acclimated in single housing for one hour. All mice were weighed prior to the experiment and single-housed for the duration of the experiment. Following CNO injection, at 30-minute, mice were live decapitated, the brain extracted from the skull, flash-frozen in liquid nitrogen, and then subsequently underwent metabolomics analysis.

### Mice

Adult (9–10 weeks old at start of experiments) KOR-Cre strain mice (RRID:IMSR_JAX:035045) were used in all experiments. Prior to the experiment, all mice were kept on a 12-hour light/12-hour dark cycle, with a room temperature of 22° ± 1°C with food and water available to them ad libitum. Throughout the ensuing testing and analysis, experimenters were blinded to the treatments given to the mice and the processing of the samples. All experimental protocols were approved by the Animal Studies Committee at Washington University.

### Surgeries

#### Chemogenetic Viral expression

At 9–10 weeks of age, experimental mice were bilaterally injected with the Gq virus, AAV5/hSyn-DIO-hm3D(Gq)-mCherry (7.8 × 1012 vg/ml, Addgene) + AAV5-EF1a-DIO-hChR2(H134R)-EYFP (2.5 × 1013 vg/ml) with a 1:1 ratio, using Neuros Syringe (65457–01, Hamilton Com.) with a volume of 100 nl on both sides of the pre-optic area (POA) (AP +0.40 mm, ML ±0.45 mm, DV-5.35 mm) with an injection speed of 30 nl/min. Control mice were injected with AAV5-EF1a-DIO-eYFP (1.8 ×1013vg/ml), with the same volume, brain coordinates and injection speed as the experimental group.

#### Body Temperature Monitoring

Two weeks following viral injection, mice were sedated with 1.5% isoflurane before implanting an iButton data temperature logger (Thermocron DS1925L#50, running OneWireViewer software, version 3.19). The skin was cut with a minor incision, and the iButton was placed at the dorsal part of the body lateral to the midline after the dermal space was opened. The mice’s skin was sutured together after the surgery using 1–2 wound surgical clips, that were then removed one week later. Alternatively, mice were implanted with wireless IPTT-300 temperature transponders (Bio Medic Data Systems; Seaford, DE) while anesthetized with 2% isoflurane. The transponder was implanted beneath the skin, rostral to the right hind leg. Three weeks following viral injection, a single test dose of CNO (clozapine-N-oxide) injection (0.5 mg/kg) was given to validate successful viral infection of POA KOR neurons as evidenced by drop in body temperature and reduced behavior activity levels. Experimental mice that did not drop their body temperature by greater than 3 degrees Celsius within 30 minutes following a CNO injection were excluded from the study.

#### Ischemic stroke

Mice underwent transient middle cerebral artery occlusion (tMCAO), as previously reported^22^. Under 2% isoflurane, the left middle cerebral artery (MCA) was exposed by a temporal muscle incision, and baseline blood flow in the cortical MCA region was measured using laser Doppler flowmetry (LDF; TSI, Inc.). A ventral midline incision was made in the neck to expose the left common carotid artery. To temporarily block the origin of the MCA, a silicon-coated, 6.0-gauge, nylon suture, measuring 12 mm in length, was inserted into the carotid artery approximately 9.0–10.5 mm in depth. A thermoregulated heating pad was used to maintain normal body temperature of 36°C during the surgical process. To restore cerebral circulation, the suture was removed after 30 minutes. Cerebral perfusion was monitored immediately after releasing the suture, and again at 72 hours. Mice that did not achieve 15% of baseline perfusion throughout the tMCAO were excluded from analysis. In addition, mice that did not achieve at least 50% of baseline flow after 5 minutes of reperfusion or 70% of baseline flow after 72 hours of reperfusion were excluded.

### Behavior

#### Rotarod

Mice were placed on a rotarod set to a constant speed of 4 rotations per minute for 5 minutes for a single trial. The time it took for the mouse to drop off the drum was recorded (maximum 300 seconds). Testing was performed before (baseline) and 72 hours after tMCAO (post 72 hours).

#### Pole

Mice were positioned with their head pointing up at the top of a 60 cm vertical pole with a 1 cm diameter. As soon as the mouse started to turn, the recording started. Recorded were the total time (Ttotal) to drop to the floor and the time (Tturn) to turn entirely to face down the pole. A maximum score was given when a mouse instantly fell off the pole. Each mouse was tested for two trials in total. The average scores of the trial one and trial two were used for data analysis. Testing was performed before (baseline) and 72 hours after tMCAO (post 72 hours).

#### Corner

Two pieces of cardboard (30 cm × 20 cm × 1 cm) were placed at a 30-degree angle to create a corner. Then the mouse was placed 1 cm away from facing the corner. A trial was recorded when the mouse moved towards the corner, and it reared in response to whisker stimulation before turning back towards the open end. The direction it turned was then recorded as left or right.

Each mouse had a total of 10 trials recorded and an asymmetry score was calculated (Right turn/(Right turn+Left turn) *100). Testing was performed before (baseline) and 72 hours after tMCAO (post 72 hours).

### Stroke Size

Seventy-two hours after tMCAO each mouse was euthanized under deep anesthesia using a full dosage of 5% isoflurane. Mice were then given an intraperitoneal euthanizing dosage of sodium pentobarbital at a concentration of 100 mg/mL per-mouse. Perfusion and isolation of the heart was done accordingly. After euthanasia and intracardiac perfusion, mice brains were removed from the skull and sliced at 1mm thickness, and subsequently stained in 2,3,5-triphenyltetrazolium chloride (TTC) at 37°C for 5 minutes. After TTC staining, each brain slice image was scanned on Adobe photoshop, and then stored in 4% paraformaldehyde. Direct infarct size in the ipsilesional side of the brain was quantified using the SigmaPlot 11.2 (Systat Software, San Jose, CA, USA) using a similar previously established protocol^22^.

### Metabolomics

Following live decapitation, whole brains were immediately removed from the skull, flash-frozen in liquid nitrogen to halt metabolism, and stored at −80°C until polar and lipid metabolite extraction as previously described^23,24^. Briefly, the brain tissue was weighed and added to a 15 mL falcon tube containing 2.8 mm ceramic beads. A 2:2:1 mixture of acetonitrile:methanol:water (40 μL per 1 mg of tissue) was added and the samples were homogenized using the tissue-specific presets on a bead mill (Omni International). Samples were incubated at −20 ° C for one hour followed by centrifugation at 10,000 RPM for 10 minutes at 4° C. The extract was transferred to autosampler vials and stored at −80° C until analysis. Ultra-high performance liquid chromatography coupled with mass spectrometry (UHPLC/MS) analyses were conducted by using a Thermo Scientific Vanquish Flex UHPLC system, interfaced with a Thermo Scientific Orbitrap ID-X Mass Spectrometer. A HILICON iHILIC-(P) Classic HILIC column (100 × 2.1 mm, 5 μm) with a HILICON iHILIC-(P) Classic guard column (20 × 2.1 mm, 5 μm) was utilized for the separation of polar metabolites. The mobile-phase solvents consisted of solvent A = 20 mM ammonium bicarbonate, 2.5 μM medronic acid, 0.1% ammonium hydroxide in 95:5 water:acetonitrile and solvent B = 2.5 μM medronic acid in 95:5 acetonitrile:water. The column compartment temperature was maintained at 45°C, and metabolites were eluted using a linear gradient at a flow rate of 250 μL/min as follows: 0–1 min, 90% B; 12 min, 35% B; 12.5–14.5 min, 25% B; 15 min, back to 90% B. For the separation of lipids, an Acquity UPLC HSS T3 column (2.1 × 150 mm, 1.8μm) with an Acquity UPLC HSS T3 VanGuard Pre-Column (2.1 × 5 mm, 1.8μm) was utilized. The mobile-phase solvents consisted of solvent A = 10 mM ammonium formate, 5 μM deactivator additive (p/n 5191–3940, Agilent Technologies) in water/acetonitrile/isopropanol (5:3:2) and solvent B = 10 mM ammonium formate, 5 μM deactivator additive (p/n 5191–3940, Agilent Technologies) in water/acetonitrile/isopropanol (5:3:2). The column compartment temperature was maintained at 60° C and lipids were eluted using a linear gradient at a flow rate of 400 μL/min as follows: 0 min 15% B, 2.5 min 50% B, 2.6 min 57% B, 9 min 70% B, 9.1 min 93% B, 11 min 96% B, 11.1 min 100% B, 12 min 100%B, 12.2 – 16 min 15% B. Data was acquired in positive and negative ion modes. The LC/MS data were then processed and analyzed using XCMS^25^, Compound Discoverer (version 3.3), and Skyline^26^. Statistical comparisons (unpaired t-tests, Benjamini and Hochberg false discovery rate <0.1), polar pathway enrichment with a hit rate threshold of ≥ 3 in MetaboAnalyst^27^, and lipid pathway analysis in BioPAN^28^.

### Statistics

Data are presented as mean ± 95% confidence interval (CI) or median with 95% CI, as appropriate. Core body temperature reductions were analyzed, based on normality assumptions, using a one-way analysis of variance (ANOVA) or Krusal-Wallis test to assess differences among the three experimental groups, followed by Tukey’s or Dunn’s multiple comparisons test for post hoc pairwise comparisons. For behavioral outcomes (rotarod, pole, and corner tests), stroke size and body weight loss, which did not meet normality assumptions, data were analyzed using the Kruskal–Wallis test, with Dunn’s multiple comparisons test for post hoc pairwise comparisons.

Mortality rates between groups were compared using Fisher’s Exact test to account for small sample sizes and categorical outcomes. Correlations between the extent of hypothermia (hypothermic index) and infarct size were evaluated at multiple time points using linear regression, reporting the coefficient of determination (R^2^) and associated p-values. All analyses and data visualization were performed using Prism (GraphPad Software, San Diego, CA). Significance was set at *P* < 0.05 unless otherwise specified.

Metabolomic data were analyzed using unpaired t-tests for comparisons between groups. A false discovery rate (FDR) correction was applied using the Benjamini–Hochberg method (threshold < 0.1) to identify statistically robust differences. Heatmaps were used to visualize global patterns of metabolite and lipid abundance, highlighting the top species showing the greatest variation across conditions. Pathway enrichment analyses were conducted to interpret the biological relevance of the identified metabolic and lipid changes. Pathway enrichment for polar metabolites was conducted using MetaboAnalyst, with a hit rate threshold >3, and lipid pathway analysis was performed in BioPAN with a significance cutoff of |Z-score| > 0.95.

## Supplementary Material

Supplement 1

Supplementary Data Figures

Materials and Methods

Supplementary Fig. S1

## Data Availability

Raw metabolomics data have been deposited in the Metabolomics Workbench (https://www.metabolomicsworkbench.org) and will be released upon publication. The accession number will be added once it is available. All other data supporting the findings of this study are included in the article and its supplementary information or is available from the corresponding author upon reasonable request.
