# Supplementary material for: Preoptic activation induces a torpor-like hypothermic and hypometabolic state that is cerebroprotective
Source: bioRxiv. 2025 Oct 25:2025.10.24.684192. Preprint. [Version 1] doi: 10.1101/2025.10.24.684192 (PMC12633306; doi:10.1101/2025.10.24.684192)
Supplement: Supplement 1 [file media-1.pdf]

Preoptic activation induces a torpor-like hypothermic and hypometabolic state that is cerebroroprotective

MATERIALS AND METHODS

Metabolic Measurements

Energy expenditure (EE), and locomotor activity (beam breaks) were assessed using a Phenomaster system (LabMaster System; TSE Systems, Bad Homburg, Germany). Mice were individually housed in metabolic cages with ad libitum access to food and water. Gases were sampled from each cage for 1 min every 10 min at a flow rate of 27 L/h. Oxygen consumption (VO_2) and carbon dioxide production (VCO_2) were measured via indirect calorimetry by comparing air from each cage to a reference cage without an animal. EE was derived from the Weir equation using constants $CVO_2 = 3.941$ and $CVCO_2 = 1.106$:

$$EE = ((CVO_2 \times VO_2) + (CVCO_2 \times VCO_2)) / 1000$$

Locomotor activity was quantified by infrared beam interruptions along the X-, Y-, and Z-axes of the cage. Activity was sampled at 100 Hz, summed in 10-min bins, and consolidated to 30-min bins. Physical activity was expressed as total beam breaks across all three axes. Prior to experimental measurements, mice were habituated in the Phenomaster cages for 3 days. For details on body temperature monitoring and DREADDs-based neuronal activation, please refer to the main Methods section of the manuscript.

RESULTS

We chemogenetically activated a subpopulation of preoptic area (POA) neurons expressing the kappa opioid receptor (KOR) and characterized the thermoregulatory, metabolic, and behavioral consequences in control (N = 7) and Hypothermic-POA^{KOR+} (N = 7) mice. Six hours post-CNO injection, Hypothermic-POA^{KOR+}mice exhibited a median core body temperature drop of −7.114 °C (95% CI of median: [−7.991, −3.270]) compared to a +0.2477 °C change in control animals (95% CI of median: [−0.9649, 0.8319]; Mann–Whitney U = 0, *P* = 0.0012, n = 6–7; Fig. S1A). At this same timepoint, energy expenditure (EE) was significantly reduced in the Hypothermic-POA^{KOR+}group (median: 8.227 kcal/hr, 95% CI of median: [7.034, 12.67]) compared to controls (median: 14.82 kcal/hr, 95% CI of median: [12.44, 16.91]; Mann–Whitney U = 1, *P* = 0.0012; Fig. S1B. Finally, locomotor activity was markedly suppressed in the Hypothermic-POA^{KOR+}group (median: 9 beam breaks, 95% CI of median: [1.000, 112.0]) compared to controls (median: 128 beam breaks, 95% CI of median: [27.00, 468.0]; Mann–Whitney U = 5, *P* = 0.0111; Fig. S1C). Thus, POA^{KOR+}neuron activation induces a hypothermic and hypometabolic state accompanied by reductions in temperature, energy use and locomotor activity.

Preoptic activation induces a torpor-like hypothermic and hypometabolic state that is cerebroroprotective

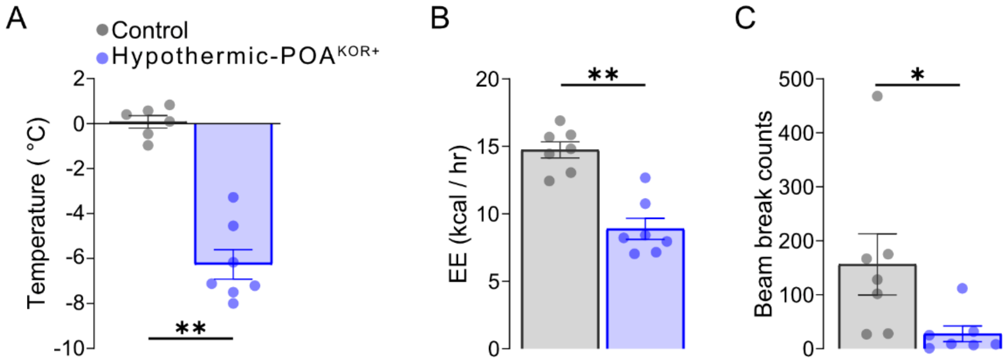

Supplementary Figure S1. Activation of POA^{KOR+} neurons induces a torpor-like hypothermic and hypometabolic state. (A) Activation of Hypothermic-POA^{KOR+} neurons led to a decrease in core body temperature 6 hours post-CNO injection. Hypothermic-POA^{KOR+}mice showed a median temperature drop of −7.114 °C (95% CI of median: [−7.991, −3.270]) from baseline, while controls showed +0.2477 °C (95% CI of median: [−0.9649, 0.8319]) (Mann–Whitney U = 0, $P = 0.0012$). (B) Energy expenditure (EE) was significantly reduced in the Hypothermic-POA^{KOR+}group (median: 8.227 kcal/hr, 95% CI of median: [7.034, 12.67]) compared to controls (median: 14.82 kcal/hr, 95% CI of median: [12.44, 16.91]) (Mann–Whitney U = 1, $P = 0.0012$). (C) Locomotor activity was significantly suppressed in Hypothermic-POA^{KOR+} mice (median: 9 beam breaks, 95% CI of median: [1.000, 112.0]) compared to controls (median: 128 beam breaks, 95% CI of median: [27.00, 468.0]) (Mann–Whitney U = 5, $P = 0.0111$). Data is shown as median ± 95% CI of the median. *$P < 0.05$, **$P < 0.01$ (two-tailed Mann–Whitney test).
